# New genomic resources for switchgrass: a BAC library and comparative analysis of homoeologous genomic regions harboring bioenergy traits

**DOI:** 10.1186/1471-2164-12-369

**Published:** 2011-07-18

**Authors:** Christopher A Saski, Zhigang Li, Frank A Feltus, Hong Luo

**Affiliations:** 1 Clemson University Genomics Institute, Clemson University, Biosystems Research Complex, 51 New Cherry Street, Clemson, SC 29634, USA; 2 Department of Genetics and Biochemisty, Clemson University, 100 Jordan Hall, Clemson, SC 29634, USA

## Abstract

**Background:**

Switchgrass, a C4 species and a warm-season grass native to the prairies of North America, has been targeted for development into an herbaceous biomass fuel crop. Genetic improvement of switchgrass feedstock traits through marker-assisted breeding and biotechnology approaches calls for genomic tools development. Establishment of integrated physical and genetic maps for switchgrass will accelerate mapping of value added traits useful to breeding programs and to isolate important target genes using map based cloning. The reported polyploidy series in switchgrass ranges from diploid (2X = 18) to duodecaploid (12X = 108). Like in other large, repeat-rich plant genomes, this genomic complexity will hinder whole genome sequencing efforts. An extensive physical map providing enough information to resolve the homoeologous genomes would provide the necessary framework for accurate assembly of the switchgrass genome.

**Results:**

A switchgrass BAC library constructed by partial digestion of nuclear DNA with *Eco*RI contains 147,456 clones covering the effective genome approximately 10 times based on a genome size of 3.2 Gigabases (~1.6 Gb effective). Restriction digestion and PFGE analysis of 234 randomly chosen BACs indicated that 95% of the clones contained inserts, ranging from 60 to 180 kb with an average of 120 kb. Comparative sequence analysis of two homoeologous genomic regions harboring orthologs of the rice *OsBRI1 *locus, a low-copy gene encoding a putative protein kinase and associated with biomass, revealed that orthologous clones from homoeologous chromosomes can be unambiguously distinguished from each other and correctly assembled to respective fingerprint contigs. Thus, the data obtained not only provide genomic resources for further analysis of switchgrass genome, but also improve efforts for an accurate genome sequencing strategy.

**Conclusions:**

The construction of the first switchgrass BAC library and comparative analysis of homoeologous harboring *OsBRI1 *orthologs present a glimpse into the switchgrass genome structure and complexity. Data obtained demonstrate the feasibility of using HICF fingerprinting to resolve the homoeologous chromosomes of the two distinct genomes in switchgrass, providing a robust and accurate BAC-based physical platform for this species. The genomic resources and sequence data generated will lay the foundation for deciphering the switchgrass genome and lead the way for an accurate genome sequencing strategy.

## Background

The global use of energy crops as renewable fuels and alternative sources of farm income is of great importance to current ecological and economic issues. Switchgrass (*Panicum virgatum*, L., Poaceae), a warm-season perennial native to the prairies of North America, has been identified by the U.S. Department of Energy (DOE) for development into an herbaceous biomass fuel crop [1,2]. Switchgrass ranges from Quebec to Central America and, like maize, is a C4 species, fixing carbon by multiple metabolic pathways with high water use efficiency [3,4]. Currently, there are breeding programs focusing on high yield, high cellulose content, and low ash characteristics [5-7]. Primarily used for summer forage, hay, and conservation plantings [8], switchgrass is an ideal biomass energy source because of its moderate to high productivity, stand longevity, high moisture and nutrient use efficiency, excellent pest and disease resistance, low cost of production, compatibility with current harvesting and haying equipment, soil restoring properties, erosion control, and adaptability to marginal soils in most agricultural regions in North America.

Given the inherent advantages of utilizing switchgrass as a biofuel feedstock, genetic improvement of switchgrass for more cost-effective biofuel conversion is a possibility that should be pursued. Feedstock traits that can be improved through breeding and biotechnology approaches include enhanced biomass yield, modified lignin production to make sugars in switchgrass more accessible, improved plant biotic and abiotic tolerance, and a long list of new potential uses for primary or co-products. All of these trait enhancements call for genomic tools development. Establishment of integrated physical and genetic maps for switchgrass will make it possible to map value added traits useful to breeding programs and to isolate important target genes using map based cloning.

Improved genetics and agronomics will certainly further enhance energy sustainability and increase biofuel yields from switchgrass. However, realizing the full genetic potential of this species is currently hampered by our lack of knowledge about its evolutionary history, closest relatives, and multiple polyploidy states. In spite of the economic and agricultural importance of switchgrass, few genomic resources exist. There are currently no reports of trait mapping in switchgrass [9], and EST and genomic microsatellite markers are just beginning to be developed [10-14]. The first publicly available switchgrass genetic map possesses only 102 RFLP markers distributed over eight homology groups [15]. A more recent work employing EST-SSR, genomic SSR and EST-STS markers led to the construction of genetic maps of two lowland switchgrass genotypes. Each map coalesced into 18 linkage groups arranged into nine homoeologous pairs [13]. Advances in sequencing and genotyping technology that have been made in the last decade will allow leapfrogging into the latest technology that should enable the application of marker assisted selection techniques such as whole genome selection. These modern breeding methods could dramatically reduce time and cost of cultivar development by reducing the amount of phenotypic analysis that is required especially in early cycles of selection. Availability of an accurate genome sequence will facilitate this vision.

It has been observed that switchgrass cultivars can have different numbers of chromosomes [16]. The reported polyploidy series in switchgrass ranges from diploid (2X = 18) to duodecaploid (12X = 108) [16-21]. To date, all lowland ecotypes examined are tetraploids (4x = 36), while upland ecotypes are tetraploids, hexaploids, or octaploids [16,20,22,23]. In 1998, DNA content (2C) for several tetraploid plants from lowland cultivars was measured and found to be 3.07 ± 0.06 pg per nucleus in average [16], so the effective genome size is ~1600 Mb for Alamo derived genotypes. To date, genomic sequencing efforts on other large, repeat-rich, plant genomes such as wheat (*Triticum estivum *L.), barley (*Hordeum vulgare *L.), maize (*Zea mays *L.), and sorghum (*Sorghum bicolor *L.) have been hindered by genomic complexity resulting in sequence assembly problems [24]. This is even more pronounced in outcrossing plants such as poplar (*Populus tremuloides *Michaux) [25]. A recent report indicated that sequencing the switchgrass genome to an 8× coverage using 2 × 150 bp of mate pair information with 454 sequencing technology would cost ~$1 million USD [26]. It has also been suggested that with this depth and type of sequencing in hand, retrotransposon families and highly repetitive regions would not be resolved well [27]. It has been suggested that a high quality physical map with enough resolution of homoeologous genomes would ensure assembly and sequencing of a minimum tiling path of large-insert clones, and the physical map would also make it possible to determine haplotypes over extensive regions [26].

Here, we present the first switchgrass BAC library with a glimpse into switchgrass genome structure and complexity as revealed by comparative sequence analysis of two homoeologous BAC assemblies derived from a genomic region that harbors orthologs of the rice *OsBRI1 *locus, a low-copy gene that encodes a putative protein kinase with a high similarity to *BRI1*, a brassinosteroid (BR) receptor in *Arabidopsis*, which has been shown to be associated with plant biomass in rice [28]. Our data support a trend toward autopolyploidy and demonstrate the feasibility of using HICF fingerprinting to resolve the homoeologous chromosomes of the two distinct genomes in switchgrass, providing a robust and accurate BAC-based physical platform for this polyploidy species. These genomic resources and sequence data will lay the foundation for deciphering the switchgrass genome and lead the way for an accurate genome sequencing strategy.

## Results

### Switchgrass BAC library construction and characterization

**Figure 1:**
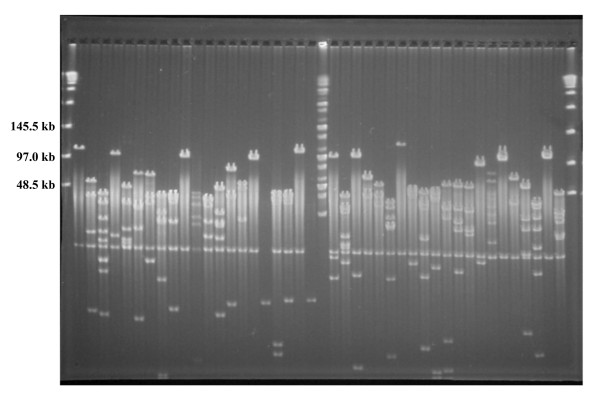
**Example insert size distribution of the switchgrass BAC library**. *Not*I digests of BAC DNA isolated from 42 random BAC clones from the switchgrass BAC library. The vector contains two *Not*I sites that flank the *Eco*RI cloning sites. The first and last lanes contain the DNA marker Lambda Ladder PFG marker (New England Biolabs, Beverly, MA) and the middle lane contains the DNA marker PFG midrange I (New England Biolabs, Beverly, MA).

**Figure 2:**
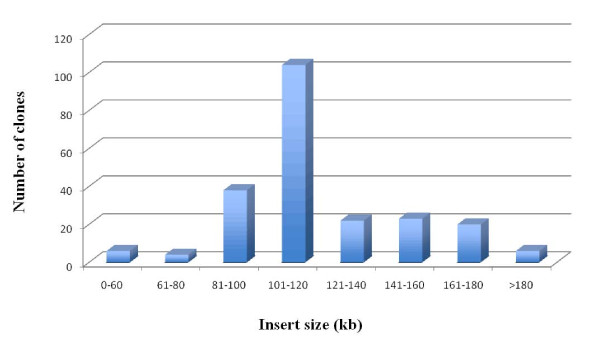
**Summary of insert size distribution of the 234 randomly chosen BAC clones**.

A BAC library of *P. virgatum *was constructed by partial digestion of nuclear DNA with *Eco*RI and contains 147,456 clones stored in 384 plates (384-well). The library covers the effective switchgrass genome approximately 10 times based on a genome size of 3.2 gigabases, and is publicly available *via *the Clemson University Genomics Institute (http://www.genome.clemson.edu). To estimate insert size and distribution of the switchgrass BAC library, 234 BACs were selected at random and analyzed by *Not*I digestion and PFGE (see example in Figure 1). Clones that appear to be partially digested were not used in insert size calculation. The results indicated that 95% of the clones contained inserts, and the insert size ranged from 60 to 180 kb with an average of 120 kb (Figure 2, Table 1).

**Table 1:** Summary of BAC library and HICF fingerprinting of BAC clones

Library	Restriction digest	Cloning vector	Average insert size (kb)	# clones	Genome coverage	Average bands/clone	# of clones fingerprinted
PV_1^a^	*Eco*RI	pCUGIBAC1	120	147,456	10 X	104^b^	18

^a^The library was constructed from *P. virgatum*

^b^Based on data from the 18 fingerprinted clones

### Identification and assembly of BACs containing switchgrass orthologs of the rice OsBRI1 gene

The genotype of the *P. virgatum *we used for BAC library construction is tetraploid. To verify whether or not switchgrass is an autopolyploid, and to examine how well the homoeologous BACs can be distinguished and correctly assembled, we set to study individual genes and their surroundings at the genome level by analyzing related BAC clones in the constructed library. To this end, we screened the BAC library with a rice *brassinosteroid insensitive 1 *(*BRI1*) homolog [28], *OsBRI1*. *BRI1 *in *Arabidopsis *and *OsBRI1 *in rice encode ubiquitously expressed putative receptor kinases. They are both single copy genes and involved in various aspects of plant growth and development. Disruption of *BRI1 *and *OsBRI1 *both led to plant dwarfism, greatly impacting plant biomass production [29-31].

**Figure 3:**
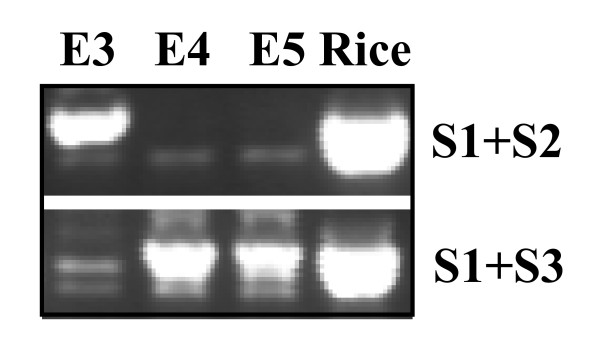
**Detection of rice *OsBRI1 *orthologs in switchgrass**. DNA from BAC clones identified by library screening with the rice *BRI1 *gene (*OsBRI1*) was used as template for PCR amplification with primers S1, S2 and S3 designed from *OsBRI1 *sequence (Table 2). Purified PCR products recovered from gel were sequenced to verify their identity. Example of PCR amplification for three orthologous BAC clones, 72H22 (E3), 72K21 (E4) and 92J12 (E5) from homoeologous chromosomes are shown.

**Figure 4:**
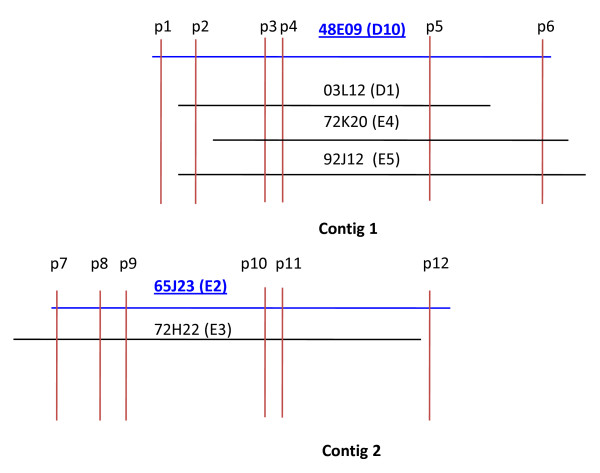
**Physical map of the switchgrass genome region containing *OsBRI1 *orthologs**. Contig assembly of the 6 *OsBRI1 *ortholog-containing BACs using HICF fingerprinting with a cutoff of e^-30 ^and a tolerance of 4 resulted in an assembly with 2 contigs: Contig 1 with 4 BACs, Contig 2 with 2 BACs. The two contigs represent two homoeologous genomes in switchgrass. The locations of the 12 predicted PCR products (p1 to p12) using the 12 pairs of primers (Additional file 1) designed based on sequence data from the two BAC clones, 48E09 (D10) and 65J23 (E2) are indicated on the map.

We screened 36,872 BAC clones with the single-copy rice gene, *OsBRI1 *that resulted in 18 positive clones, 6 of which showed strong signal intensity and 12 showed moderate to weak signal strength. To further confirm that the 6 clones showing strong hybridization signals with *OsBRI1 *probe contain switchgrass orthologs of *OsBRI1 *gene, two pairs of PCR primers were designed based on sequence of the conserved region of the rice *OsBRI1 *gene. PCR analysis of the switchgrass *OsBRI1 *homolog using DNA from the 6 identified BAC clones resulted in two distinct amplification patterns. The clones E2 and E3 gave rise to a PCR product with the primer pair of S1, S2, but not with the primer pair of S1, S3. On the contrary, the clones D1, D10, E4 and E5 gave rise to a PCR product with the primer pair of S1, S3, but not with the primer pair of S1, S2 (see example in Figure 3). Sequencing of the two PCR products indicated that they are different *OsBRI1 *orthologs with high homology (data not shown). Sequencing of representative BAC clones from both groups (see next section) revealed differences in various positions of the S1, S2 and S3 primers between *OsBRI1 *and its switchgrass counterparts in two BACs (Table 2). It is therefore tempting to speculate that switchgrass *OsBRI1 *orthologs are single-copy genes, residing in the two homoeologous genomes in the autotetraploid switchgrass, and the two groups of BACs represent DNA fragments from homoeologous regions surrounding *OsBRI1 *orthologs. To examine this possibility, all 18 identified BACs were subsequently subjected to restriction patterning to determine the extent of shared overlap by High Information Content Fingerprinting (HICF) [32-34]. BAC fingerprinting assembly at 1e^-70^, tolerance of 4, resulted in three contigs, of which two corresponded to the two different groups of clones initially revealed by PCR analysis of the *OsBRI1 *orthologs, i.e., D1, D10, E4 and E5 were in contig 1, whereas E2 and E3 were in contig 2 (Figure 4). A third contig, Contig 3 was composed of 4 BACs identified with moderate hybridization signals that did not produce any positive PCR amplicons when analyzed using primer pairs of S1, S2 and S1, S3 respectively.

**Table 2:** Comparison of primer sequences between *OsBRI1 *and its switchgrass homologs^a^

Primer	S1	S2	S3
Rice	TGGAGACCATTGGCAAGATC	GCGATTTTCAAATGCTCCAG	TCCAAGCACGCACAGGCG
BAC 48E09 (D10) ^b^	TGGAGACTATTGGCAAGATC	GCAACCTTCAAGTGCTCCAG	TCCAAGCAAGCACAAGCA
BAC 65J23 (E2) ^b^	TGGAGACCATTGGCAAGATC	GCAACCTTCAGGTGCTCCAG	TCCAGGCAAGCACAAGCA

^a^Primers were designed based on sequence of the hypothetically conserved region of the rice *OsBRI1 *gene

^b^The corresponding sequences of the switchgrass homologs of the *OsBRI1 *gene in two homoeologous BACs are also listed. The different nucleotides are underlined.

### BAC sequencing and assembly

To gain insight into switchgrass *OsBRI1 *orthologs and their surrounding sequences in the two homoeologous genomes, a consensus BAC from each of the two mapped fingerprint contigs, contig 1 and contig 2 (Figure 4) was sequenced to an 8× coverage, assembled, and finished according to the bridging shotgun method and the Bermuda standards for finishing. Sequencing of the BAC 48E09 (D10) from the contig 1, and 65J23 (E2) from the contig 2 resulted in a total length of 122,250 bp and 102,051 bp, respectively. Sequence data from the two *OsBRI1*-containng BAC representatives not only allow for comparative genomic analysis of the switchgrass genome but also provide references to validate assembly of contigs and evaluate switchgrass genome using additional molecular approaches.

### Validation of the two switchgrass homoeologous contigs spanning the regions containing the OSBRI1 orthologs

**Figure 5:**
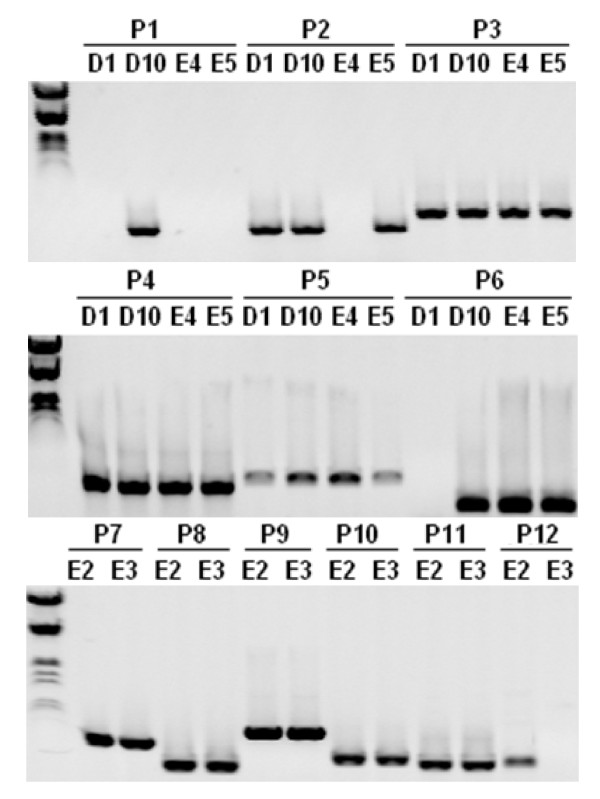
**PCR assessment for validation of the *OSBRI1 *ortholog-containing contigs representing two homoeologous genomes in switchgrass**. Ethidium bromide-stained agarose gel showing PCR products amplified from DNAs of the six BAC clones assembled in the two contigs as shown in Figure 4. Primers designed for PCR amplification of DNA fragments (p1 to p12) as indicated in Figure 4 were listed in Additional file 1. A molecular weight ladder is shown on the left. Results of PCR amplification with 12 primer pairs for the 6 BAC clones were summarized in Table 3.

To confirm correct chromosomal locations and genome assignments of the 6 switchgrass BAC clones assembled into two homoeologous contigs spanning the regions containing the *OSBRI1 *orthologs (Figure 4), twelve pairs of primers were designed based on the sequences of the two BAC clones, 48E09 (D10) and 65J23 (E2), and utilized for PCR with DNAs from the 6 *OsBRI1 *ortholog-containing BACs (Additional file 1). Presence or absence of PCR amplicons in each of the 6 BACs mapped to the two contigs should confirm correct contig assembly, and therefore the genome and homoeologous chromosomal assignment. As demonstrated in Figure 5 and summarized in Table 3, the presence or absence of PCR amplicons for each of the 6 BACs match the patterns as predicted from the physical maps, thus supporting contig assembly obtained by BAC fingerprinting (Figure 4). These initial data indicate the feasibility of using HICF fingerprinting to resolve the homoeologous chromosomes of the two distinct genomes in switchgrass, providing a robust and accurate BAC-based physical platform for this polyploidy species.

**Table 3:** Summary of PCR assessment for contig validation (see Figure 5)^a^

Primer Pairs	D1	D10	E2	E3	E4	E5
p1	-	+	-	-	-	-
p2	+	+	-	-	-	+
p3	+	+	-	-	+	+
p4	+	+	-	-	+	+
p5	+	+	-	-	+	+
p6	-	+	-	-	+	+
p7	-	-	+	+	-	-
p8	-	-	+	+	-	-
p9	-	-	+	+	-	-
p10	-	-	+	+	-	-
p11	-	-	+	+	-	-
p12	-	-	+	-	-	-

^a^"+" denotes PCR positive, whereas "-" denotes PCR negative

### Assessment of the tetraploid switchgrass genome by Southern analysis

**Figure 6:**
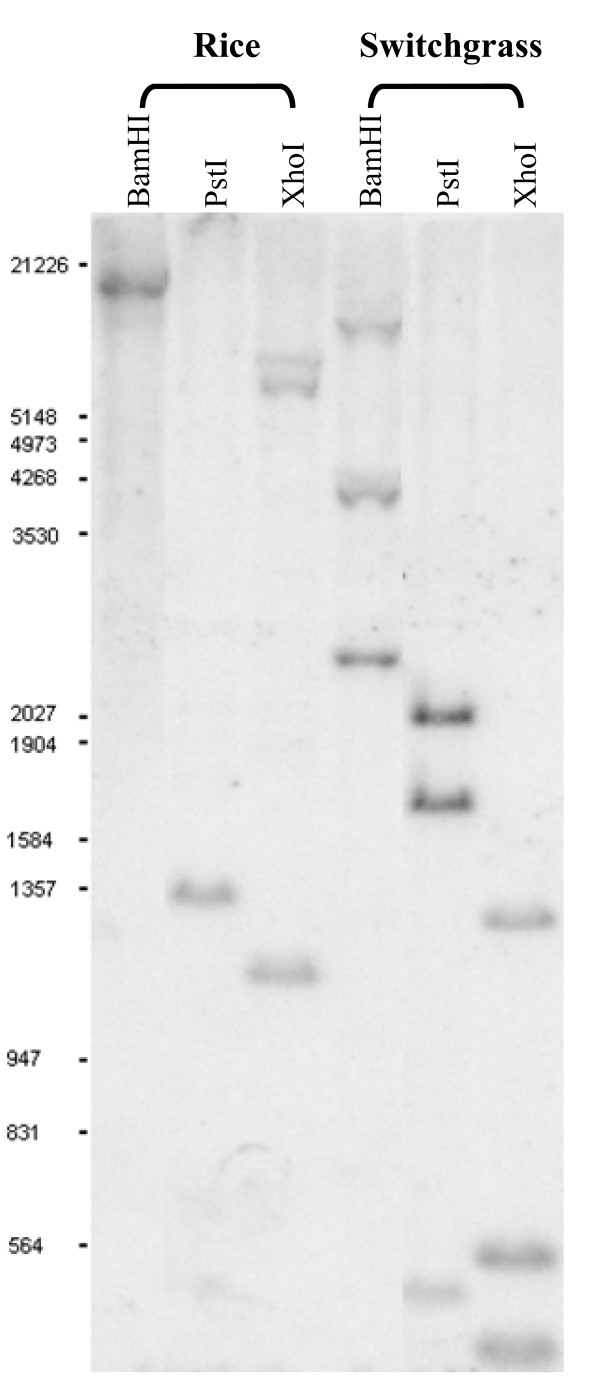
**Southern analysis of switchgrass genome region containing *OSBRI1 *homolog**. The genomic DNA of rice and switchgrass plants was digested with *Bam*HI, *Pst*I and *Xho*I respectively and the *OsBRI1 *gene was used as the probe for hybridization with the digested DNA. Predicted and observed RFLP patterns revealed by *OSBRI1 *in rice and switchgrass were summarized in Table 4.

Sequencing data from the two switchgrass *OsBRI1 *ortholog-containing BACs provide information about restriction sites surrounding the *OsBRI1 *orthologs. To further confirm that orthologs of the *OSBRI1 *are indeed single-copy genes in switchgrass, we conducted southern analysis of switchgrass genomic DNA using the rice *OsBRI1 *gene as probe. DNA was digested with three enzymes, *Bam*HI, *Pst*I and *Xho*I, and rice genomic DNA was used as positive control. Hybridization of *OsBRI1 *to *Bam*HI- or *Pst*I-digested rice genomic DNA both resulted in one single band of expected size as predicted from rice genomic DNA sequence (Figure 6, Table 4). When *Xho*I-digested rice genomic DNA was probed by *OsBRI1*, two bands of expected sizes (~1.2 kb and 5.4 kb) were revealed (Figure 6, Table 4). An additional band of > 6 kb was also observed most probably due to incomplete digestion of genomic DNA (Figure 6). Similarly, switchgrass genomic DNA digested with various restriction enzymes led to the same patterns as predicted from the sequences of the two BAC clones, and no additional hybridization signals were detected (Figure 6, Table 4). Hybridization of *Bam*HI-digested genomic DNA with heterologous probe, *OsBRI1 *revealed three bands of ~2.3 kb, 7.3 kb, and 7.8 kb as well as an additional band of > 3 kb (~ 4.0 kb), in agreement with the pattern predicted from the sequences surrounding the *OsBRI1 *orthologs in the two BAC clones, 48E09 (D10) and 65J23 (E2) (Figure 6, Table 4). Hybridization of *Pst*I-digested genomic DNA with heterologous probe, *OsBRI1 *revealed three bands of ~2.0 kb, 1.7 kb and 0.5 kb as expected (Figure 6, Table 4), one additional predicted DNA fragment of 88 bp was not observed in this blot because it had run out of the gel during electrophoresis, which was confirmed in a separate Southern hybridization experiment (data not shown). In the case of DNA digested by *Xho*I, Southern hybridization resulted in three bands of predicted sizes of ~1.3 kb, 0.6 kb and 0.45 kb (Figure 6, Table 4). The fourth band of 382 bp had run out of the gel during electrophoresis, which was confirmed in a separate Southern hybridization experiment (data not shown). Thus, Southern analysis results strongly suggest that the switchgrass *OsBRI1 *orthologs exist as single-copy genetic loci, residing in two homoeologous genomes, and that the tetraploid switchgrass is autopolyploid.

**Table 4:** Predicted and observed RFLP patterns revealed by *OSBRI1 *in rice and switchgrass (see Figure 6)

Clone (bp)	Probe position (bp)	*Bam*HI		*Pst*I		*Xho*I	
	
		Expected (bp)	Actual (bp)	Expected (bp)	Actual (bp)	Expected (bp)	Actual (bp)
Switchgrass BAC 48E09 (122250)	73868	2339	+	523	+	382	*
	-	7758	+	1673	+	446	+
	75557					589	+
						1308	+

Switchgrass BAC 65J23 (102051)	98980	7304	+	88	*	382	*
	-	> 3000	~4000	2023	+	446	+
	100669					589	+
						1308	+

Rice PAC AP003453 (151100)	66842	10590	+	94	*	1162	+
	-			304	*	5438	+
	68522			1367	+		

*DNA fragments smaller than 400 bp that have run out of gel during electrophoresis

### Homoeology and extent of fractionation between switchgrass BACs

**Figure 7:**
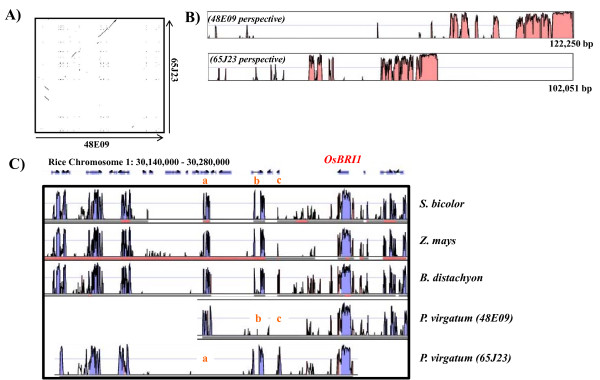
**Intra-/Inter-genomic alignment of switchgrass orthologous regions**. (**A**) Blastz alignment dot plot of switchgrass BACs (48E09 = 122,250 bp; 65J23 = 102,051 bp). (**B**) VISTA percent identity plot of switchgrass BACs from both BAC perspectives. (**C**) Alignment of switchgrass BACs and other grasses to VISTA determined orthologous rice genome region. The hybridization probe, *OsBRI1*, is shown in red. Putatively fractionated genes are shown in orange (a = LOC_Os1g51980; b = LOC_Os1g52010; c = LOC_Os1g52030).

The two switchgrass BACs sequenced in this study were identified based upon their hybridization to the same *OsBRI1 *probe and position in different BAC fingerprint contigs (Figure 4). Those experiments do not provide sequence level evidence that these two BACs are indeed homoeologous (polyploidy derived paralogy) and are derived from two ancestral genomes. The BAC to BAC alignment can be visualized in Figure 7A and 7B as a dot plot or percent identity plot, respectively. Using all 35 BLASTN [35] alignments greater than 50 bp in length, 18,189 nucleotides overlap between the two BACS with an average percent identity of 94%. Both switchgrass BACs map to the same orthologous region in rice (see "Methods") suggesting that they came from a common ancestral position (Figure 7C). In addition, the repeat density for these two BACs is highly similar (48E09 = 25.9%; 65J23 = 25.4%; Additional file 2) also suggesting that they come from similar genomic regions.

## Discussion

A critical tool for the genomic enablement of any plant species with a large, complex genome is the availability of a deep-coverage, large-insert (BAC) library that can serve as the centrepiece for the generation and integration of genome-level information. BAC libraries serve as the platform for physical mapping, positional cloning, integration of genetic maps, comparative studies, and whole-genome sequencing. We constructed the first switchgrass BAC library consisting of 147,456 clones with an average insert size of 120 kb using genomic DNA from a lowland ecotype, Alamo derived genotype that has been demonstrated to be superior in biomass production (Wu, personal communication). The switchgrass BAC library totals 10 × coverage of the total nuclear content of this tetraploid switchgrass genotype and 5 × coverage of each of the two genomes.

Polyploids are common in seed plants with the majority of them being allopolyploids. Polyploidy plays an important role in plant genome evolution [36-38]. It has been suggested that the tetraploid switchgrass lowland ecotypes behave as an autopolyploid with complete or near complete disomic inheritance and specific bivalent pairing of chromosomes in groups of four at meiosis [15]. Our data using two sequenced BAC clones suspected to reside on homoeologous genomes, support a trend toward autopolyploidy and the feasibility of resolving the homoeologous genomes in switchgrass using high information content fingerprinting. Saini et al. [39] demonstrated this when the polyploidy nature of *Glycine max *was assessed by BAC-based fingerprinting, integration of genetic markers, and whole-genome/BAC-end sequence. In soybean, 5-10% sequence divergence is necessary to separate the homoeologs by HICF fingerprinting [39]. This group used BAC-end sequences from three minimum tile paths to examine the extent of polyploidy-like regions within contigs, and the extent of correlation between the polyploidy-like regions inferred from fingerprinting and the polyploid-like sequences inferred from whole genome shotgun sequence matches [39]. The result improved whole genome sequence contig and scaffold building methods by sequence assessment and physical location. In order to fully understand the origins of the polyploid switchgrass, whole genome sequencing of related taxa and fluorescent in situ hybridization techniques should be used.

In an effort to assess the possibility of BAC-based physical mapping in polyploidy plant species, Luo et al. [24] assembled contigs of fingerprinted clones in an *in silico *merged BAC library composed of single-chromosome libraries of two wheat homoeologous chromosome arms, 3AS and 3DS, and complete chromosome 3B. An average of 97.78% or more clones from a single chromosome arm was achieved, indicating a very high fidelity of contig assembly and the negligibly low level of incorporation of clones from homoeologous chromosome arms into a contig during contig assembly. This result strongly suggests the feasibility of contig construction and physical mapping using global BAC libraries in polyploidy species.

In the current project, we identified 18 clones positive for the *OsBR11 *homologue by DNA hybridization to the BAC filters. Using HICF, we assembled a BAC clone based physical map of the rice homologue *OsBRI1 *genomic region in switchgrass and sequenced a consensus BAC from each of the homoeologous contigs for comparative analysis. Of the 18 positive clones, 6 and 12 showed strong and moderate signal intensity, respectively. Upon further analysis of the homoeologous contigs, both switchgrass BACs map to the same orthologous region in rice suggesting that they came from a common ancestral position. The degree of intragenomic alignment and preservation of gene order relative to all the four grass genomes suggest that the 2 BACs are homoeologous yet have undergone significant fractionation. The degree of fractionation can be qualitatively seen in the alignment of orthologous regions in rice and three additional grasses (Figure 7C). For example, it appears that the sub-genome from which 48E09 (D10) was derived has lost at least two rice genes (LOC_Os1g52010, LOC_Os1g52030) whereas these genes were maintained in the genome from which 65J23 (E2) was derived yet this genome has lost LOC_Os1g51980. These comparative genomic data, degree of sequence divergence, and physical map evidence suggest that the two switchgrass genomes are similar enough to discover homoeologous segments yet divergent enough to make it difficult to select indistinguishable BACs from a BAC library.

To our knowledge, this is the very first report using one gene, *OsBRI1 *to comprehensively characterize switchgrass genome organization from various aspects demonstrating the feasibility of using HICF to resolve the homoeologous chromosomes of the two distinct genomes in this species. Next step would be to extend the analysis using multiple loci covering large genomic regions to further validate the data presented in this study.

BAC-based whole genome physical mapping has recently evolved into the second generation arena and advancement in resolution (i.e., single nucleotide resolution vs. restriction patterning) suggests more robust contig builds. For example, van Oeveren et al. studied the feasibility of using a next-generation sequencing-based strategy, the whole-genome profiling (WGP) for physical mapping [40]. De novo contigs assembly by WGP technology in *Arabidopsis thaliana *ecotype Columbia resulted in high quality physical map, which was confirmed using the Columbia reference sequence. The application and simulation of this technology in other plant genomes seem to suggest its scalability to complex genomes [40]. Although it remains unclear if next generation physical mapping can resolve homoeologous genomic segments, high-throughput next-generation sequencing-based technologies could potentially lead to more robust solutions towards highly reliable physical map construction, therefore worth exploring. In either case, a heuristic BAC by BAC sequencing approach from a minimum tiling path of the switchgrass genome should work.

## Conclusions

We have constructed a large insert BAC library from a tetraploid switchgrass genotype that contains about 10 genome equivalents with an average insert size of 120 kb. The feasibility of contigs assembly, and eventually physical map construction from fingerprinted BAC clones in a polyploid species was studied. Results from comparative analysis of BACs containing orthologs of the rice *OsBRI1 *locus demonstrated that orthologous clones from homoeologous chromosomes can be unambiguously distinguished from each other, and correctly assembled to respective contigs. Thus, the data obtained from this research not only provide genomic resources and sequence data for further analysis of switchgrass genome, but also lead the way for an accurate genome sequencing strategy.

## Methods

### Plant material

The Alamo derived tetraploid switchgrass genotype, SL93 2001-1, kindly provided by Dr. Yanqi Wu (Oklahoma State University, Stillwater, OK, USA) was grown and maintained in the greenhouse at Clemson University.

### BAC library construction and characterization

The plants were kept in dark conditions 24 hours prior to harvesting. Approximately 50 grams of young, expanding leaf tissues were harvested and immediately flash frozen in liquid nitrogen prior to nuclei preparation. Nuclei purification and embedding, *Eco*RI partial restriction enzyme digestion of DNA, as well as the preparation of high molecular weight DNA fragments were conducted following Luo and Wing [41]. Preparation of the *Eco*RI cloning-ready single copy pIndigoBAC536 vector from the high copy pCUGIBAC1 plasmid was performed according to Luo et al. [42]. The size-selected high molecular fragments were ligated to vector and transformed into *E. coli *strain DH10B (Invitrogen, Carlsbad, CA, USA). White recombinant colonies were selected on LB plates containing chloramphenicol, X-Gal, IPTG, and picked robotically using the Genetix Q-bot (Genetix, UK). Recombinant clones were transferred into individual wells of 480, 384-well microtiter plates, grown and then stored at -80°C. The BAC library was gridded onto 10, 11.25 × 22.25 cm filters in high density, double spots (18,432 clones represented per filter) and 4 × 4 patterns. To characterize the BAC inserts, BAC DNA was prepared according to standard alkaline lysis conditions in a 96-well format, digested with *Not*I, and separated by pulsed field gel electrophoresis (PFGE) on a 1% agarose gel with the following conditions: 5-15 sec linear ramp time, 6 V/cm, 14°C in 0.5 × TBE buffer for 15 hours and stained with ethidium bromide.

### PCR analysis of BAC clones

PCR analysis was conducted to analyze BAC clones. The primers used were listed in Table 2 and Additional file 1. The reaction mixtures (25 μl total volume) consisted of 50 mM KCl, 10 mM Tris-HCl (pH 8.8), 1.5 mM MgCl_2_, 0.1% (w/v) Triton X-100, 200 μM each of dATP, dCTP, dGTP, and dTTP, 0.5 μM of each primer, 0.2 μg of template DNA, and 1 unit of Taq DNA polymerase (Promega, Madison, WI, USA). Amplification was performed in a Bio-Rad MJ mini gradient thermal cycler (Hercules, CA, USA) programmed for 25 cycles of 1 min at 94°C (denaturation), 2 min at 55°C (hybridization), 3 min at 72°C (elongation) and a final elongation step at 72°C for 10 min. PCR products were separated in a 0.8% (w/v) agarose gel and detected by staining with ethidium bromide.

### Southern analysis of genomic DNA

Plant genomic DNA was isolated from about 300 to 500 mg young leaves using the cetyltrimethyl ammonium bromide (CTAB) method, essentially as described by Luo et al. [43]. Ten micrograms of DNA were digested with *Bam*HI, *Pst*I and *Xho*I after the supplier's instruction (New England Biolabs, Beverly, MA, USA). Digested DNA was fractionated through a 0.7% (w/v) agarose gel and blotted on to a Hybond-N^+ ^filter (GE Healthcare Bio-Sciences Corp., Piscataway, NJ, USA). The 622 bp fragment of *OsBRI1 *gene amplified from rice genomic DNA with primers, S1 and S2 as described above in Table 2 were used as probe. Probe was labelled with the [α-^32^P]dCTP using Prime-It II Random Primer Labeling Kit (Stratagene, La Jolla, CA, USA). DNA blot was probed in Church buffer at 65°C and exposed on phosphor screens at RT overnight and scanned using Typhoon 9400 (GE Healthcare Bio-Sciences Corp., Piscataway, NJ, USA).

### Identification of switchgrass BACs containing OsBRI1 homologue

The switchgrass BAC library was screened to identify *OsBRI1 *ortholog-containing clones following the procedure described previously [44]. Briefly, approximately 25 ng of the *OsBRI1 *PCR amplicon was radiolabelled with ^32^P-dATP using the DECAprime™ random priming DNA labelling kit (Ambion, Inc). Labelled probe was denatured and added against the A and B filters of the switchgrass BAC library. Hybridization was performed at 60°C overnight. Filters were washed with 1 × SSC, 0.1% SDS at 60°C for 30 minutes for 5 times and exposed to phosphor screens, and the images were recorded by a Typhoon 9400 Imager (GE Healthcare Bio-Sciences Corp., Piscataway, NJ, USA). The addresses of the positively hybridized BAC clones were manually scored.

### Physical map of the OsBRI1 orthologs region in switchgrass homoeologues

We performed HICF on the 18 strong and moderate positively identified BAC clones and experimented with a range of assembly parameters in FPC to identify the most commensurate build. The parameters ranged from a cutoff of e^-30^, to e^-70^, and a tolerance of 4. The first assembly at 1e-^70^, tolerance of 4 resulted in 3 contigs: Contig 1 with 3 BACs, Contig 2 with 2 BACs, and Contig 3 with 3 BACs, and 10 singletons. The FPC assembly using a cutoff of e^-30 ^and a tolerance of 4 resulted in a similar assembly with 3 contigs: Contig 1 with 4 BACs, Contig 2 with 2 BACs, and Contig 3 with 4 BACs, and 8 singletons. It is likely that the BACs not assembling into a respective contig is due to a small amount of overlap, stringent assembly conditions, or non-specific identification during DNA hybridization.

### Small-insert library production and sequencing of homoeologues

Small-insert DNA fragments were generated by hydroshearing BAC DNA isolated as a maxi-prep from the BAC clone. Fragments between 3 and 5 kb were size selected by gel electrophoresis, end-repaired, cloned into the high copy plasmid based cloning vector pBlueskriptIIKSII+, and then electroporated into *E. coli *DH10B cells. Transformants were selected on LB plates containing carbenicillin, X-Gal and IPTG. White recombinant colonies were picked robotically using the Genetix Q-bot and stored as individual clones in Genetix 96-well microtiter plates as glycerol stocks at -80°C. Sub-clones were sequenced with the universal priming sites on the vector in both the forward and reverse directions and sequence data collected on an ABI 3730xl DNA Analyzer. Sequence data was collected to an 8 × sequence coverage.

### Inter- and intra-genome comparisons

Switchgrass BACs were aligned to each other using either blastz at the Pipmaker [45] server (http://pipmaker.bx.psu.edu/pipmaker/) or mVISTA [46] server (http://genome.lbl.gov/vista/) using the LAGAN [47] algorithm. The orthologous region of each switchgrass BAC in *Oryza sativa *(v5.0) was determined using the GenomeVISTA server (http://genome.lbl.gov/vista/). These overlapping orthologous regions were then compared with the VISTA pre-computed orthologous region for *Zea mays *(v.3), *Sorghum bicolor *(v.1.0) and *Brachypodium distachyon*. Switchgrass repeat component was determined using the Censor [35,48] server (http://www.girinst.org/censor/) and *Poaceae *repeat libraries.

## Authors' contributions

CAS constructed and screened the library, conducted HICF physical mapping and BAC sequencing. ZL conducted PCR analysis of BAC clones and Southern analysis. FAF conducted comparative genomic analysis of the *OsBRI1 *ortholog-containing BACs. CAS and HL drafted the manuscript. HL conceived, initiated and supervised the project. All authors read and approved the final manuscript.

## Supplementary Material

Additional file 1 **Primers designed for use in PCR analyses presented in Figure 5 to verify and validate the contig assembly**. Click here for file

Additional file 2 **Repeat density of the two BACs, 48E09 (D10) and 65J23 (E2)**. Click here for file
